# Impact of CDX2 expression status on the survival of patients after curative resection for colorectal cancer liver metastasis

**DOI:** 10.1186/s12885-018-4902-8

**Published:** 2018-10-16

**Authors:** Yasuyuki Shigematsu, Kentaro Inamura, Noriko Yamamoto, Yoshihiro Mise, Akio Saiura, Yuichi Ishikawa, Shunji Takahashi, Hiroaki Kanda

**Affiliations:** 1 Department of Pathology, The Cancer Institute of Japanese Foundation for Cancer Research (JFCR), 3-8-31 Ariake, Koto, Tokyo, 135-8550 Japan; 2 Division of Gastroenterology Center, The Cancer Institute Hospital, JFCR, 3-8-31 Ariake, Koto, Tokyo, 135-8550 Japan; 3 Division of General Oncology, The Cancer Institute Hospital, JFCR, 3-8-31 Ariake, Koto, Tokyo, 135-8550 Japan; 4 Department of Pathology, Saitama Cancer Center, 780 Komuro, Ina, Kita-adachi-gun, Saitama, 362-0806 Japan

**Keywords:** CDX2, Colorectal cancer, Liver metastasis, Metastasectomy, Chemotherapy

## Abstract

**Background:**

The prognostic biomarker for patients undergoing curative liver metastasectomy for colorectal cancer (CRC) is lacking. The purpose was to investigate the prognostic role of a lack of CDX2 expression, which has been proposed as a potential biomarker for high-risk relapse in early-stage CRC, in patients undergoing curative liver metastasectomy.

**Methods:**

A total of 396 consecutive patients with CRC liver metastasis who underwent potentially curative liver metastasectomy at a single center in Japan between 2005 and 2015 were included. For the immunohistochemical analysis of nuclear CDX2 expression, we adopted the tissue microarray approach using the resected metastatic liver CRCs. Patient subgroups were compared with respect to disease-free survival (DFS) and overall survival (OS) by applying the Kaplan-Meier curve, log-rank tests, and multivariate analyses based on the Cox proportional hazards method. OS is defined as the period from the date of curative liver resection for metastatic CRC until death. DFS is defined as the length of time from curative liver resection to either the first recurrence or death. In patients without recurrence, the latest imaging inspection date was used as the censored date.

**Results:**

Thirty-six of the 396 CRCs (9.1%) reduced CDX2 expression. The reduced expression of CDX2 was associated with poor differentiation (*P* = 0.02). DFS in days was lower in the patients with CDX2-low CRC than in the patients with CDX2-high CRC (median DFS: 245 days versus 420 days; hazard ratio for disease recurrence: 1.64; 95% confidence interval: 1.08–2.38; *P* = 0.02). OS in days was lower in the patients with CDX2-low CRC than in the patients with CDX2-high CRC (median OS: 1024 days versus 3145 days; hazard ratio: 2.41; 95% confidence interval: 1.52–3.85; *P* <  0.001). In patients with CDX2-low CRC, both DFS and OS were similar between the with and without pre- or post-operative chemotherapy groups (median DFS: 243 versus 247 days; *P* = 0.73, median OS: 1016 versus 1363 days; *P* = 0.69).

**Conclusions:**

Reduced expression of CDX2 indicates poor DFS and OS, however, it might not represent chemosensitivity in patients undergoing curative liver metastasectomy. (339/350).

**Electronic supplementary material:**

The online version of this article (10.1186/s12885-018-4902-8) contains supplementary material, which is available to authorized users.

## Background

The most effective treatment option available for patients with resectable colorectal cancer (CRC) liver metastasis is surgical resection. However, the recurrence rate after this procedure is 50–70%, and this practice alone is insufficient in managing the disease. Although supplementing the surgical resection with chemotherapy is considered to improve the overall curability, there is no strong evidence of its efficacy [1, 2]. The heterogeneity of patients with resectable CRC liver metastasis in terms of prognosis is one of the reasons for the difficulty in obtaining proof for the chemotherapeutic benefit in clinical trials. To select high-risk patients with resectable CRC liver metastasis, there is a need for a marker to predict poor prognosis.

In a large retrospective study, the absence of CDX2 expression, which regulates gastrointestinal epithelial development and maturation [3–6], was proposed as a poor prognostic and predictive biomarker for the response to chemotherapy in stages II and III CRC [7]. In addition, in stage IV unresectable CRC, the lack of CDX2 expression reportedly predicted poor survival [8]. However, the previous studies did not focus on patients undergoing metastasectomy [9, 10], and the prognostic and predictive implications of a deficient CDX2 expression in patients who underwent curative resection for CRC liver metastasis remain unclear.

Several features of the CDX2 expression are involved in CRC. First, the expression is heterogeneous. Second, this heterogeneity in the primary site remains in liver metastasis, and the metastatic process does not affect the expression status. Third, the consistency in CDX2 expression between the primary and metastatic sites is not affected by chemotherapy [11]. Along with these unique features, previous studies also revealed that the lack of CDX2 expression is associated with poor differentiation, right-sided occurrence, *BRAF* mutation, CpG island methylator phenotype positivity, and microsatellite instability [9, 10, 12]. On the other hand, as a clinical behavior, the recurrence pattern of CRCs lacking CDX2 expression after the liver metastasectomy is currently unknown.

Therefore, we performed a retrospective analysis to investigate the value of the reduced expression of CDX2 as a potential prognostic biomarker for patients undergoing potentially curative liver metastasectomy. In addition, we assessed the difference in the recurrence behavior between without and with CDX2 expression in CRC. To this end, we adopted the tissue microarray (TMA) approach for efficiently handling a large sample size. To cover the expression heterogeneity, three locations per tumor were analyzed according to the previous TMA studies for CRC [13, 14].

## Methods

### Patients and tissue samples

In this retrospective study, CRC surgical samples were obtained from 396 consecutive patients wtih adenocarcinoma who underwent curative liver resection for metastatic CRC at the Cancer Institute Hospital in Tokyo, Japan, between 2005 and 2015. All the samples were collected from surgically resected liver metastatic lesions. The clinical data and histological features including age, sex, tumor location, number of liver metastases, initial clinical-stage, chemotherapy history, *KRAS* mutation status, and tumor grade were obtained from the electronic medical records. Pathological studies were performed by three experienced pathologists (Y.S., K.I., and H.K.) while conforming to the fourth edition of the World Health Organization criteria [15]. The tumor stage was determined according to the seventh edition of the tumor-node-metastasis staging manual of the American Joint Committee on Cancer [16]. Patients who received chemotherapy within 6 months before or after the metastasectomy were defined as those with a history of pre- or post-operative chemotherapy. We excluded patients for whom adequate tissue samples, follow-up data, or written informed consent was lacking. The study was approved by the ethical committee of the Cancer Institute Hospital (No. 2016–1087).

### Patient outcome

The survival status of the 396 patients was assessed until December 2017. The primary outcome measure was overall survival (OS), which is defined as the period from the date of potentially curative liver resection for metastatic CRC until death. The secondary outcome was the disease-free survival (DFS), defined as the length of time from potentially curative liver resection to either the first recurrence or death. Contrast-enhanced computed tomography and magnetic resonance imaging were employed for evaluating the recurrence, and the inspection date was taken as the recurrence confirmation date. In patients without recurrence, the latest imaging inspection date was used as the censored date.

### Tissue microarrays

The archived surgically resected specimens, which had been subjected to the initial pathological diagnosis of metastatic CRC, were exploited for the construction of TMAs. We punched points on the donor paraffin blocks using a 2-mm-diameter coring needle and transferred the tissue to the array in the recipient block using a manual tissue arrayer (KIN; Azumaya, Tokyo, Japan), as described previously [17]. For each tumor, three tissue cores of a 2-mm-diameter site depicting the tumor’s most representative histology were selected by experienced liver pathologists (Y.S. and H.K.).

### Immunohistochemical analysis

#### CDX2 expression

**Fig. 1:**
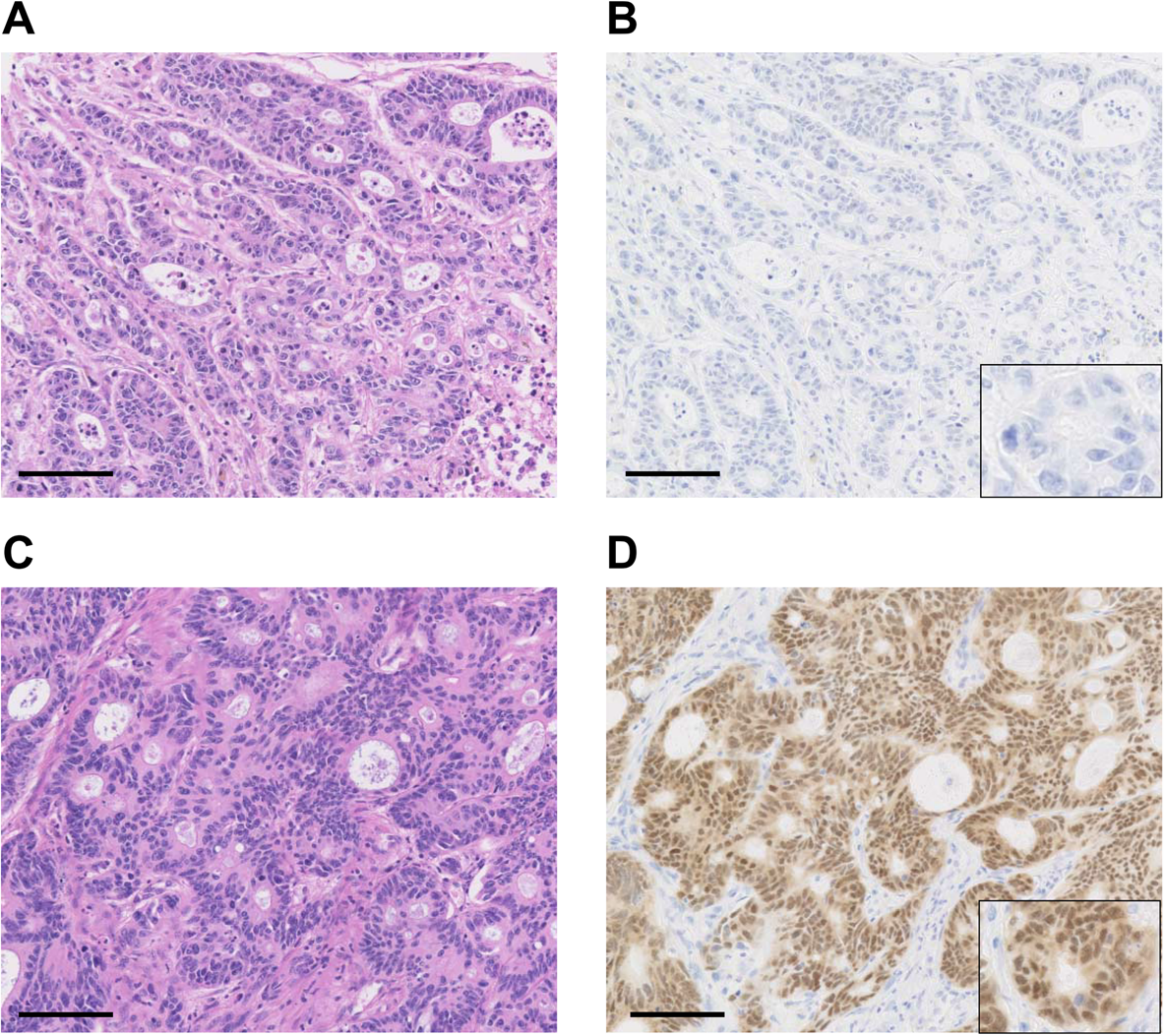
Representatives of CDX2-low and CDX2-high colorectal cancer (CRC). **a** and **b** CDX2-low CRC. **c** and **d** CDX2-high CRC. Left images **a** and **c**: Hematoxylin and eosin staining. Right image **b** and **d**: CDX2 immunohistochemistry. Scale bar = 100 μm

Immunohistochemistry (IHC) was performed using 4-μm thick formalin-fixed paraffin-embedded (FFPE) TMA sections. A mouse antihuman CDX2 monoclonal antibody (clone DAK-CDX2, 1:100 dilution, DAKO, Carpinteria, CA, USA) was used as the primary antibody. All samples were handled in an anonymous fashion. The tissue slides were stained using a Bond-III automated stainer (Leica Microsystems, Buffalo Grove, IL, USA), and antigen detection was achieved using the Bond Polymer Refine Detection Kit (Leica Microsystems). We evaluated the CDX2 IHC in terms of nuclear expression (Fig. 1) and regarded a tissue core of CRC as CDX2-low when the tumor completely lacked or possessed < 50% of CDX2-positive cancer cells. We determined the CDX2 expression status of the tumor based on the status of the majority of the tissue cores. We referred normal colonic epithelial cells and non-epithelial cells as positive and negative controls for CDX2 nuclear expression, respectively. All cases were scored blindly by experienced pathologists (Y.S. and K.I.) in an independent manner and those that were difficult to interpret immunohistochemically were discussed with a third expert (H.K.).

#### Mismatch repair protein expression

IHC for the assessment of mismatch repair protein expression was conducted by using the following primary monoclonal antibodies: mouse antihuman MLH1 (clone ES05, 1:100; Leica Biosystems, Newcastle, UK), mouse antihuman MSH2 (clone G219–1129, 1:500; BD Biosciences, Tokyo, Japan), MSH6 (clone EPR3945, 1:200; GeneTex, Tokyo, Japan), and PMS2 (clone A16–4, 1:100; BD Biosciences, Tokyo, Japan). The loss of a mismatch repair protein was defined as the absence of nuclear expression in the tumor cells with a positive nuclear expression of the lymphocytes or the epithelium. Three tissue cores per tumor were studied for each protein expression analysis. We considered a tumor to have lost its protein expression when all the three tissue cores did not express the protein. Deficient mismatch repair protein (dMMR) was defined as the expression loss of a tumor’s mismatch repair protein. Two pathologists (Y.S. and K.I.) independently evaluated the protein expression in this case.

### Statistical analysis

Statistical analyses were conducted using R version 3.2.2 (R Foundation for Statistical Computing, Vienna, Austria). The continuous features were summarized as the mean, median, and range, while the categorical features were summarized as frequencies and percents. The comparison of the clinical and histological aspects between the patients with CDX2-low and CDX2-high CRC was performed using the Mann-Whitney U test, Fisher’s exact test, and Student’s *t*-test. Cohen’s kappa index was applied to examine the concordance of the CDX2 expression status determined by the two investigators. OS and DFS were estimated using the Kaplan-Meier method. Patients with CDX2-low and CDX2-high CRC were compared with respect to the survival outcomes using the Kaplan–Meier curves, log-rank tests, and multivariate analyses based on the Cox proportional hazards method. Two-tailed *P* values of less than 0.05 were considered statistically significant.

## Results

### Clinicopathological features of CDX2-low CRCs

A total of 36 CDX2-low samples were identified among the 396 CRCs (prevalence: 9.1%; 95% confidence interval [CI]: 6.4–12.3%). Of the 36 CDX2-low CRCs, 13.9% were poorly differentiated adenocarcinoma, which is quite high when compared with only 3.6% in the CDX2-high CRCs (*P* = 0.02; Table 1). In this study, we did not find a statistically significant difference in the sex and tumor location between the patients with CDX2-low and CDX2-high CRCs (*P* = 0.58), while the association with a lack of CDX2 expression was previously reported [8, 9]. Moreover, even in terms of the incidence of liver metastasis, which is an indicator of tumor aggressiveness, we did not perceive a statistically significant difference between the CDX2-low and CDX2-high CRCs (average number of metastasis; 4.9 versus 4.5, *P* = 0.68). Of the 36 and 360 individuals with CDX2-low and CDX2-high CRCs, 25 (69.4%) and 246 (68.3%) patients, respectively, underwent pre- or/and post operative systemic chemotherapy for the liver metastasis (most patients received FOLFOX [folinic acid, 5-fluorouracil, and oxaliplatin] with or without bevacizumab or cetuximab).

**Table 1:** Clinicopathological characteristics between CDX2-high and CDX2-low colorectal cancer

Variable	Total (*n* = 396)	CDX2 expression		*P* value
		High (*n* = 360)	Low (*n* = 36)	
Age^a^				
≦ 65	234 (59.1)	211 (58.6)	23 (63.9)	0.60
65 <	162 (40.9)	149 (41.4)	13 (36.1)	
Sex^a^				
Female	136 (34.3)	122 (33.9)	14 (38.9)	0.58
Male	260 (65.7)	238 (66.1)	22 (61.1)	
Histology^a^				
Well	63 (15.9)	56 (15.6)	7 (19.4)	0.02
Mod	315 (79.5)	291 (81.1)	24 (66.7)	
Por	18 (4.5)	13 (3.6)	5 (13.9)	
Tumor location of the primary site^a^				
Right	93 (23.5)	82 (22.8)	11 (30.6)	0.56
Left	155 (39.1)	143 (39.7)	12 (33.3)	
Rectum	148 (37.4)	135 (37.5)	13 (36.1)	
Number of metastasis^b^	4.5 (± 6.3)	4.5 (± 6.3)	4.9 (± 5.5)	0.68
Liver metastasis^a^				
Synchronous	223 (56.3)	207 (57.5)	16 (44.4)	0.16
Metachronous	173 (43.7)	153 (42.5)	20 (55.6)	
Pre- or post-operative chemotherapy^a^				
No	125 (31.6)	114 (31.7)	11 (30.6)	1.00
Yes	271 (68.4)	246 (68.3)	25 (69.4)	

Abbreviations: *well* Well differentiated, *mod* Moderately differentiated, *por* Poorly differentiated

^a^Data presented as n (%)

^b^Data presented as mean (± standard deviation)

To assess the reproducibility of our interpretation of the CDX2 expression status, concordance among the CDX2 interpretation results of all the CRCs obtained by two independent investigators were analyzed, which revealed a rate of 93.9% (Kappa index, 0.84; *P* <  0.001). These results suggest that our evaluation of the CDX2 expression is robust.

### CDX2 expression status, DFS, and OS of patients who received curative liver metastasectomy

**Fig. 2:**
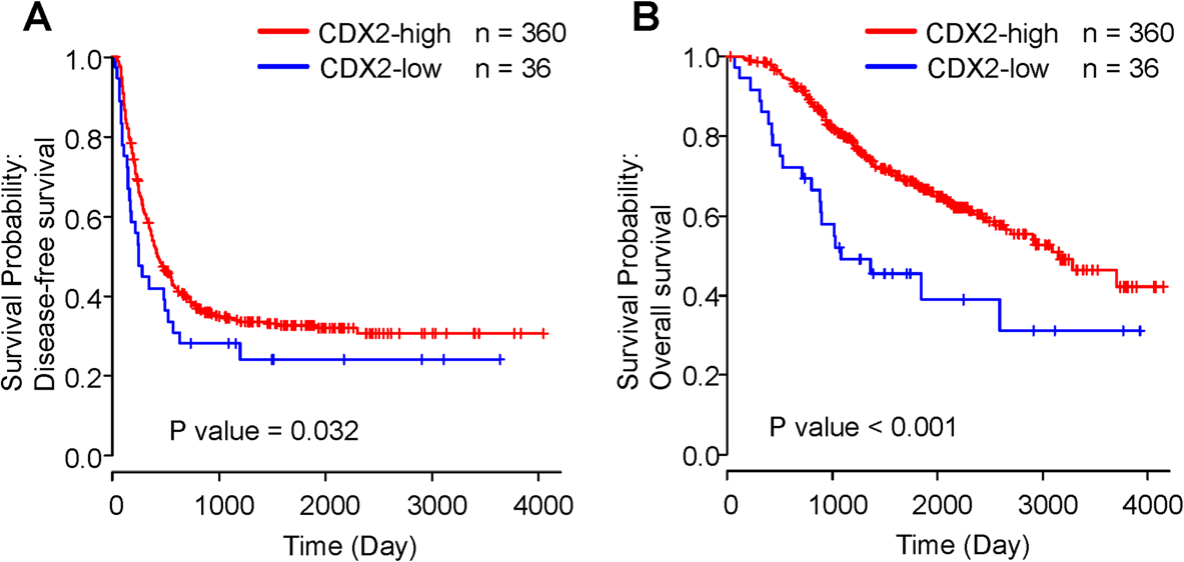
Kaplan–Meier curves of disease-free survival (DFS) and overall survival (OS). **a** The association of CDX2 expression with DFS in patients undergoing potentially curative liver metastasectomy. **b** The association of CDX2 expression with OS in patients undergoing potentially curative liver metastasectomy. Blue and red lines represent CDX2-low and CDX2-high CRC, respectively

The median DFS in days for the CDX2-low and CDX2-high patients who underwent liver metastasectomy were 245 and 420 days, respectively (*P* = 0.032; Table 2; Fig. 2a; Additional file 1: Table S1). In the multivariate analysis that included sex, chemotherapy history, as well as tumor grade, tumor location, and synchronous or metachronous liver metastasis as the confounding variables, the hazard ratio (HR) for disease recurrence in the patients with CDX2-low versus those with CDX2-high was 1.64 (95% CI, 1.08–2.38; *P* = 0.02; Table 3). The reduced expression of CDX2 expression was the only remaining variable. As the post-relapse treatment, seven patients (2.7%) received best supportive care due to poor performance status or the patient’s wishes, 159 patients (61.1%) underwent metastasectomy with curative intent, and 94 patients (36.2%) received palliative chemotherapy.

**Table 2:** Relationship between CDX2 expression and survival in patients after potentially curative liver metastasectomy

Survival (days)	CDX2 expression		Univariate HR (95% CI)	*P* value
	High	Low		
Median DFS	420	245	1.53 (1.03–2.26)	0.032
Median OS	3145	1024	2.45 (1.55–3.86)	< 0.001

*HR* Hazard ratio, *OS* Overall survival, *DFS* Disease-free survival

**Table 3:** Multivariate model to predict DFS and OS in patients after potentially curative liver metastasectomy

Features	DFS		OS	
	HR (95% CI)	*P* value	HR (95% CI)	*P* value
CDX2 expression				
High	1 (reference)		1 (reference)	
Low	1.64 (1.08–2.38)	0.02	2.41 (1.52–3.85)	< 0.001
Sex				
Female	1 (reference)		1 (reference)	
Male	0.96 (0.73–1.24)	0.74	0.94 (0.65–1.34)	0.74
Tumor grade				
Well	1 (reference)		1 (reference)	
Mod	1.04 (0.73–1.47)	0.83	1.29 (0.79–2.10)	0.30
Por	1.24 (0.68–2.28)	0.48	1.73 (0.73–4.04)	0.21
Pre- or post-operative chemotherapy				
No	1 (reference)		1 (reference)	
Yes	0.92 (0.68–1.22)	0.55	1.58 (1.06–2.36)	0.02
Tumor location				
Right	1 (reference)		1 (reference)	
Left	0.85 (0.62–1.17)	0.33	0.67 (0.44–1.01)	0.06
Rectum	0.97 (0.71–1.37)	0.97	0.63 (0.68–1.05)	0.08
Liver metastasis				
Synchronous	1 (reference)		1 (reference)	
Metachronous	0.59 (0.45–0.77)	< 0.001	1.25 (0.88–1.79)	0.20

Abbreviations: *well* Well differentiated, *mod* Moderately differentiated, *por* Poorly differentiated, *HR* Hazard ratio, *OS* Overall survival, *DFS* Disease-free survival

The median OS in days for the CDX2-low and CDX2-high patients who underwent the metastasectomy were 1024 and 3145 days, respectively (*P* <  0.001; Table 2; Fig. 2b; Additional file 1: Table S1). The poor prognostic impact of the reduced expression of CDX2 on the OS remained significant in multivariate analyses that included sex, tumor grade, and synchronous or metachronous liver metastasis as the confounding variables (HR 2.41; 95% CI, 1.52–3.85; *P* <  0.001; Table 3). These findings specify that the CDX2-low CRC is associated with a poor prognosis than CDX2-high CRC, even in patients who underwent curative resection for the liver metastases.

In an exploratory analysis, focusing on the patients undergoing preoperative chemotherapy alone, we evaluated the association between CDX2 expression and DFS or OS. A total of 12 CRCs with CDX2-low were identified among the 79 CRCs. The median DFS in days for the CDX2-low and CDX2-high patients were 195 and 361 days, respectively, and the difference was not statistically significant (*P* = 0.39; Additional file 2: Figure S1A). The median OS in days for the CDX2-low patients was significantly worse than the CDX2-high patients (761 versus 2344 days; *P* = 0.0019; Additional file 2: Figure S1B). In the multivariate analysis that included sex, chemotherapy history, tumor grade, tumor location, and synchronous or metachronous liver metastasis as the confounding variables, the CDX2 expression status was remaining variable and the HR for overall survival in the patients with CDX2-low versus those with CDX2-high was 3.38 (95% CI, 1.54–7.41; *P* = 0.002; Additional file 3: Table S2).

As for the patients undergoing postoperative chemotherapy alone, a total of five CRCs with CDX2-low were identified among the 93 CRCs. The median DFS in days for the CDX2-low and CDX2-high patients were 1202 and 747 days, respectively (*P* = 0.803; Additional file 2: Figure S1C). Likewise, OS in days for the CDX2-low and CDX2-high patients were similar between the two groups (*P* = 0.89; Additional file 2: Figure S1D). These data indicate the prognostic impact of CDX2 expression status might differ between preoperative and postoperative patients.

### CDX2 expression status and sensitivity to pre- or post-operative chemotherapy

**Fig. 3:**
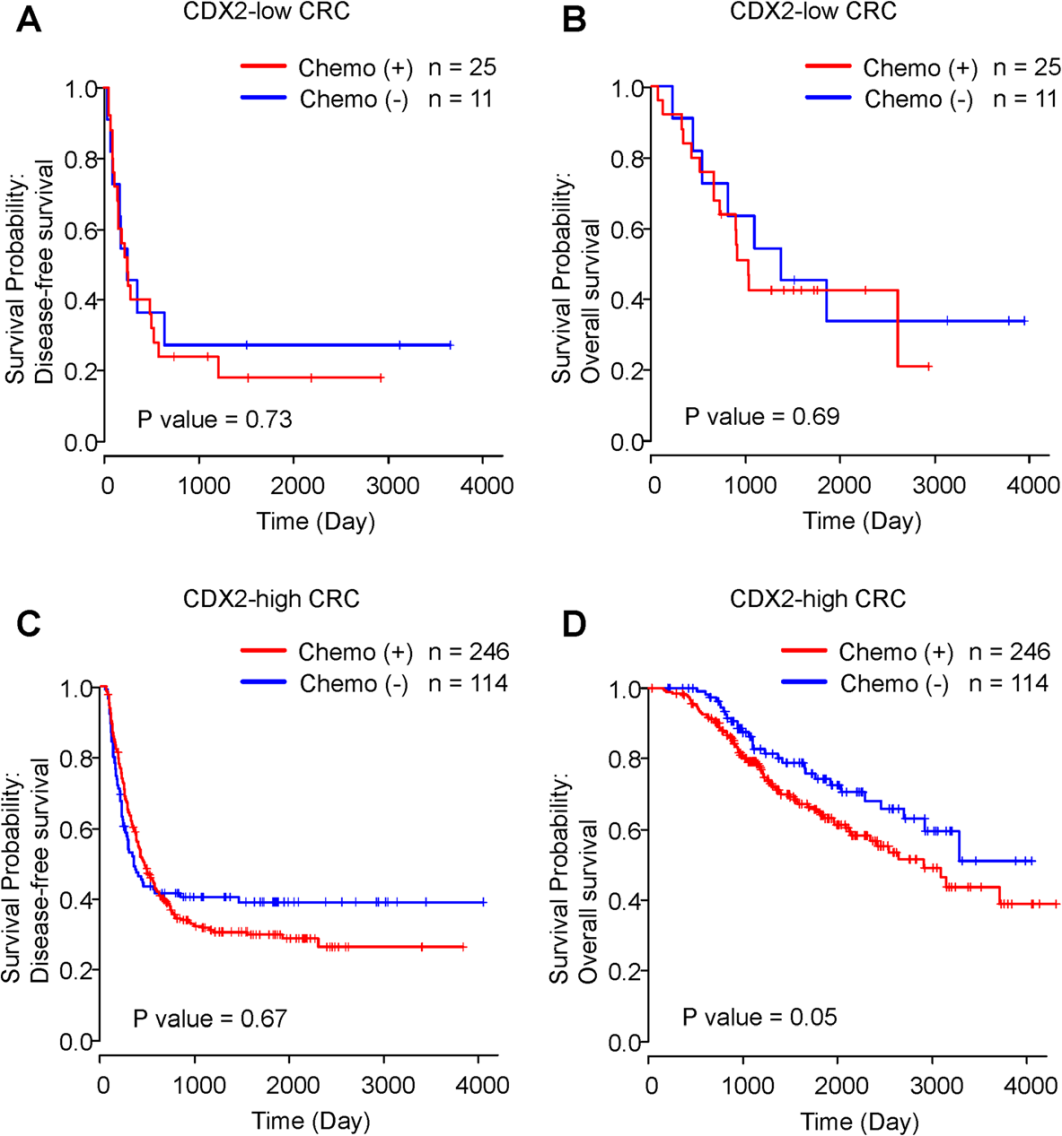
Kaplan–Meier curves of disease-free survival (DFS) and overall survival (OS) in patients without and with pre- or post-operative chemotherapy. **a** and **b** The association of pre- or post-operative chemotherapy with DFS and OS in patients with CDX2-low CRC. **c** and **d** The association of pre- or post-operative chemotherapy with DFS and OS in patients with CDX2-high CRC. Red and blue lines represent patients with and without pre- or post-operative chemotherapy, respectively

To investigate the impact of CDX2 expression status on the sensitivity to chemotherapy, we assessed DFS and OS in patients with CDX2-low CRC who either did or did not receive pre- or post-operative chemotherapy for liver metastasectomy. In the preliminary test, substantial differences were not observed between the with and without pre- or post-operative chemotherapy for CDX2-low CRC in terms of DFS and OS in days (DFS: 243 versus 247 days; *P* = 0.73, OS: 1016 versus 1363 days; *P* = 0.69, Fig. 3a, b). The patients with CDX2-high CRC, too did not receive considerable survival benefits from pre- or post-operative chemotherapy (median DFS: 457 versus 348 days; *P* = 0.67, median OS: 2908 days versus not available; *P* = 0.054, Fig. 3c, d). These results signify that the CDX2 expression status might not strongly predict the survival benefits of chemotherapy in patients who had undergone liver metastasectomy.

### Difference in the recurrence pattern between patients with CDX2-low and CDX2-high CRC after the liver metastasectomy

To evaluate the difference in recurrence sites between the CDX2-low and CDX2-high CRCs, we explored the first recurrence site in patients with CRC liver metastasis after the curative resection. Of the 396 patients with recurrence after the liver metastasectomy, 260 (65.7%) faced recurrence. The recurrence rates in the CDX2-low and CDX2-high CRCs were 28 (77.8%) and 232 (64.4%), respectively. The recurrence sites included liver (74.2%), lung (23.1%), peritoneum (3.8%), adrenal gland (0.8%), brain (0.8%), bone (0.8%), and ovary (0.4%). Interestingly, no significant difference in recurrence sites was observed between the CDX2-low and CDX2-high CRCs (Additional file 4: Table S3).

Among the 260 recurrence patients, 222 (85.4%), 32 (12.3%), and 4 (1.5%) experienced recurrence in one, two, and three organs, respectively, when the recurrence was firstly detected. The number of recurrence organs in the patients with CDX2-low CRC was significantly higher than in the patients with CDX2-high CRC (*P* <  0.001; Table 4). These observations demonstrate that CDX2-low CRCs metastasize to the other organs more easily than CDX2-high CRCs.

**Table 4:** Number of sites of recurrence in patients with CDX2-high and CDX2-low colorectal cancer after potentially curative liver metastasectomy

Number of sites of recurrence	Total (*n* = 260)	CDX2 expression		*P* value
		High (*n* = 232)	Low (*n* = 28)	
1	222 (85.4)	205 (89.1)	17 (60.7)	< 0.001
2	32 (12.3)	22 (9.6)	10 (35.7)	
3	4 (1.5)	3 (1.3)	1 (3.6)	

Data presented as n (%)

### Molecular features of CDX2-low CRCs in patients undergoing the liver metastasectomy

As a secondary analysis, to evaluate the molecular features of CDX2-low CRCs, we examined the association between the dMMR and CDX2 expression status. The prevalence of dMMR in patients who underwent curative resection was 14 (3.5%; 95% CI: 2.0–5.9%). The frequencies of the dMMR presence in CDX2-low and -positive CRCs were eight (22.2%) and six (1.7%), respectively, and the CDX2-low CRCs were significantly associated with the presence of dMMR (*P* <  0.001; Additional file 5: Table S4).

As for the *KRAS* status, the number of patients whose status could be accessed was 174. Among them, the *KRAS* mutation was prevalent in 57 patients (32.8%; 95% CI: 25.8–40.3%). The frequencies of the *KRAS* mutation in the CDX2-low and CDX2-high CRCs were 2 (14.3%) and 55 (34.4%), respectively, with no significant difference between the two groups (*P* = 0.14; Additional file 6: Table S5).

## Discussion

This retrospective study proved that the patients with CDX2-low CRC who underwent potentially curative liver metastasectomy had poor DFS and OS than those with CDX2-high CRC. In addition, we unraveled the uniqueness of the recurrence pattern in CDX2-low CRCs when compared with CDX2-high CRCs. To the best of our knowledge, this is the first report that establishes the prognostic impact of CDX2 expression status on patients undergoing potentially curative liver metastasectomy as well as establishes the difference in the recurrence patterns between the CDX2-low and CDX2-high CRCs.

Appropriate patient risk stratification in terms of prognosis is crucial for ensuring the best pre- or post-operative long-term oncological outcomes. The lack of CDX2 expression is associated with a poor prognosis in stages II, III, and unresectable stage IV CRC [7, 8]. However, the prognostic impact of the absent CDX2 expression in patients undergoing liver metastasectomy remains poorly understood. In this study, we analyzed the CDX2 expression of CRC in 396 patients receiving the procedure and discovered that the reduced expression of CDX2 was linked to poor prognosis even in patients who received curative liver metastasectomy. This effect was independent of several known risk factors, including the pathological grade.

Although previous studies reported an association between the lack of CDX2 expression and high malignancy potential [9, 10], the difference in recurrence pattern between CRC with and without CDX2 expression was hitherto unknown. Our study confirmed that the number of recurrence organs in CDX2-low CRC was higher than that in CDX2-high CRC. Conversely, the number of recurrent liver tumors, where recurrence was most frequently observed after liver metastasectomy, was similar between the two groups. These findings indicate that the CDX2-low CRC has a higher metastatic potential, which leads to a poor prognosis. Hence, a wide range of organs should be screened for recurrence especially in patients with CDX2-low CRC, even after the potentially curative resection.

The sensitivity of CDX2-low CRC to chemotherapy remains controversial. In contrast to the reported benefit of adjuvant chemotherapy in stages II and III CDX2-low CRC [7], the advantage of systemic chemotherapy in unresectable metastatic CRC appeared to be less [8]. As for patients undergoing curative liver metastasectomy, we observed almost similar survival curves (DFS and OS) for with and without pre- or post-operative chemotherapy in patients having CDX2-low CRC. The patients who underwent pre- or post-operative chemotherapy included borderline resectable and initially unresectable cases. Without the pre- or post-operative chemotherapy, their prognoses are considered to be worse than the initially resectable cases [18–20]. Thus, it is possible that the borderline or initially unresectable cases may attain survival rates similar to the resectable ones due to the chemotherapeutic approach for taking CDX2-low CRC. Due to the selection bias, the results should be further validated by randomized prospective studies.

In our Japanese population study, CDX2-low CRC was associated with poor differentiation and dMMR. These observations are consistent with those of the previous studies from Europe and North America [21–24] and the area differences in molecular features of CDX2-low CRC appear to be negligible. Considering that the CDX2-low CRCs are also reportedly associated with several adverse prognostic variables [9, 10], such as advanced stage, vascular invasion, *BRAF* mutation, and CIMP-positive status, the absence of CDX2 expression alone might signify the presence of multiple biological risk factors.

Our study has several limitations. First, we employed TMA approach for evaluating the protein expression of CRC and assessed a limited area. Although the CDX2 expression exhibited heterogeneity in CRC [11], we selected three tissue cores to cover the heterogeneity. Along with the previous studies having stated that equal to or more than three tissue cores per CRC can reflect the whole section in terms of IHC [13, 14], the effects elicited by the TMA’s disadvantages are trivial. Second, the number of patients with CDX2-low CRC is relatively small. To obtain a clear conclusion regarding the CDX2-low CRC’s sensitivity to chemotherapy and the association between the timing of perioperative chemotherapy and the prognostic impact of CDX2 expression, further studies with a large sample size are necessary. Third, the information on the *KRAS* mutation status was limited. In the current study, data on the mutation were available only for 43% of the patients because health insurance did not cover the examination of the *KRAS* mutation and its status had been analyzed until 2010. However, we believe that selection bias was negligible because the prevalence of the mutation and the results of the association between the CDX2 expression status and *KRAS* were similar to previous studies [25–28].

## Conclusions

Our study has demonstrated for the first time that the reduced expression of CDX2 in CRC is a poor prognostic factor for patients undergoing potentially curative liver metastasectomy, and this effect is independent of pre- or post-operative chemotherapy. On the other hand, the CDX2 expression status might not represent chemosensitivity for those patients.

## Additional files

Additional file 1: **Table S1.** Univariate analysis to investigate a relation between each parameters and survival. (DOC 86 kb)

Additional file 2: **Figure S1.** Kaplan-Myere curves of disease-free survival (DFS) and overall survival (OS) in patients undergoing either preoperative or postoperative chemotherapy. (A, B) Those in patients undergoing preoperative chemotherapy. (C, D) Those in patients undergoing postoperative chemotherapy. Blue and red lines represent CDX2-low and CDX2-high CRC, respectively. (TIFF 623 kb)

Additional file 3: **Table S2.** The relationship between CDX2 expression and overall survival in patients undergoing preoperative chemotherapy. (DOC 80 kb)

Additional file 4: **Table S3.** Site of recurrence in patients with CDX2-high and CDX2-low colorectal cancer after potentially curative liver metastasectomy. (DOC 78 kb)

Additional file 5: **Table S4.** Association between mismatch repair-deficient and CDX2 expression status in patients with colorectal cancer after potentially curative liver metastasectomy. (DOC 53 kb)

Additional file 6: **Table S5.** Association between *KRAS* and CDX2 expression status in patients with colorectal cancer after potentially curative liver metastasectomy. (DOC 53 kb)

## Abbreviations

CI: Confidence interval
CRC: Colorectal cancer
DFS: Disease-free survival
dMMR: Deficient mismatch repair protein
FFPE: Formalin-fixed paraffin embedded
HR: Hazard ratio
IHC: Immunohistochemistry
OS: Overall survival
TMA: Tissue microarray
