# Supplementary material for: Impact of CDX2 expression status on the survival of patients after curative resection for colorectal cancer liver metastasis
Source: BMC Cancer. 2018 Oct 16;18:980. doi: 10.1186/s12885-018-4902-8 (PMC6192098; doi:10.1186/s12885-018-4902-8)
Supplement: Supplementary file 1 — Table S1. Univariate analysis to investigate a relation between each parameters and survival. (DOC 86 kb) [file 12885_2018_4902_MOESM1_ESM.doc]

| **Additional file 1: Table S1. Univariate analysis to investigate a relation between each parameters and survival.** | | | | | | |
| --- | --- | --- | --- | --- | --- | --- |
| Features |  | DFS | |  | OS | |
|  |  | HR (95% CI) | P value |  | HR (95% CI) | P value |
|  |  |  |  |  |  |  |
| CDX2 expression |  |  |  |  |  |  |
| High |  | 1 (reference) |  |  | 1 (reference) |  |
| Low |  | 1.51 (1.03 – 2.25) | 0.036 |  | 2.46 (1.56 – 3.89) | <0.001 |
|  |  |  |  |  |  |  |
| Sex |  |  |  |  |  |  |
| Female |  | 1 (reference) |  |  | 1 (reference) |  |
| Male |  | 0.96 (0.74 - 1.24) | 0.75 |  | 0.92 (0.65 - 1.30) | 0.64 |
|  |  |  |  |  |  |  |
| Tumor grade |  |  |  |  |  |  |
| Well |  | 1 (reference) |  |  | 1 (reference) |  |
| Mod |  | 1.09 (0.77 - 1.54) | 0.61 |  | 1.28 (0.80 - 2.07) | 0.30 |
| Por |  | 1.53 (0.84 - 2.78) | 0.15 |  | 2.21 (0.97 - 5.06) | 0.058 |
|  |  |  |  |  |  |  |
| Perioperative chemotherapy |  |  |  |  |  |  |
| No |  | 1 (reference) |  |  | 1 (reference) |  |
| Yes |  | 0.96 (0.70 - 1.22) | 0.58 |  | 1.45 (0.99 - 2.12) | 0.053 |
|  |  |  |  |  |  |  |
| Tumor location |  |  |  |  |  |  |
| Right |  | 1 (reference) |  |  | 1 (reference) |  |
| Left |  | 0.86 (0.63 - 1.17) | 0.33 |  | 0.62 (0.42 - 0.94) | 0.023 |
| Rectum |  | 0.94 (0.69 - 1.28) | 0.69 |  | 0.66 (0.43 - 0.99) | 0.048 |
|  |  |  |  |  |  |  |
| Liver metastasis |  |  |  |  |  |  |
| Synchronous |  | 1 (reference) |  |  | 1 (reference) |  |
| Metachronous |  | 0.62 (0.48 - 0.80) | <0.001 |  | 1.15 (0.82 - 1.60) | 0.42 |
|  |  |  |  |  |  |  |
