# Supplementary figures and images for: Impact of CDX2 expression status on the survival of patients after curative resection for colorectal cancer liver metastasis
Source: BMC Cancer. 2018 Oct 16;18:980. doi: 10.1186/s12885-018-4902-8 (PMC6192098; doi:10.1186/s12885-018-4902-8)

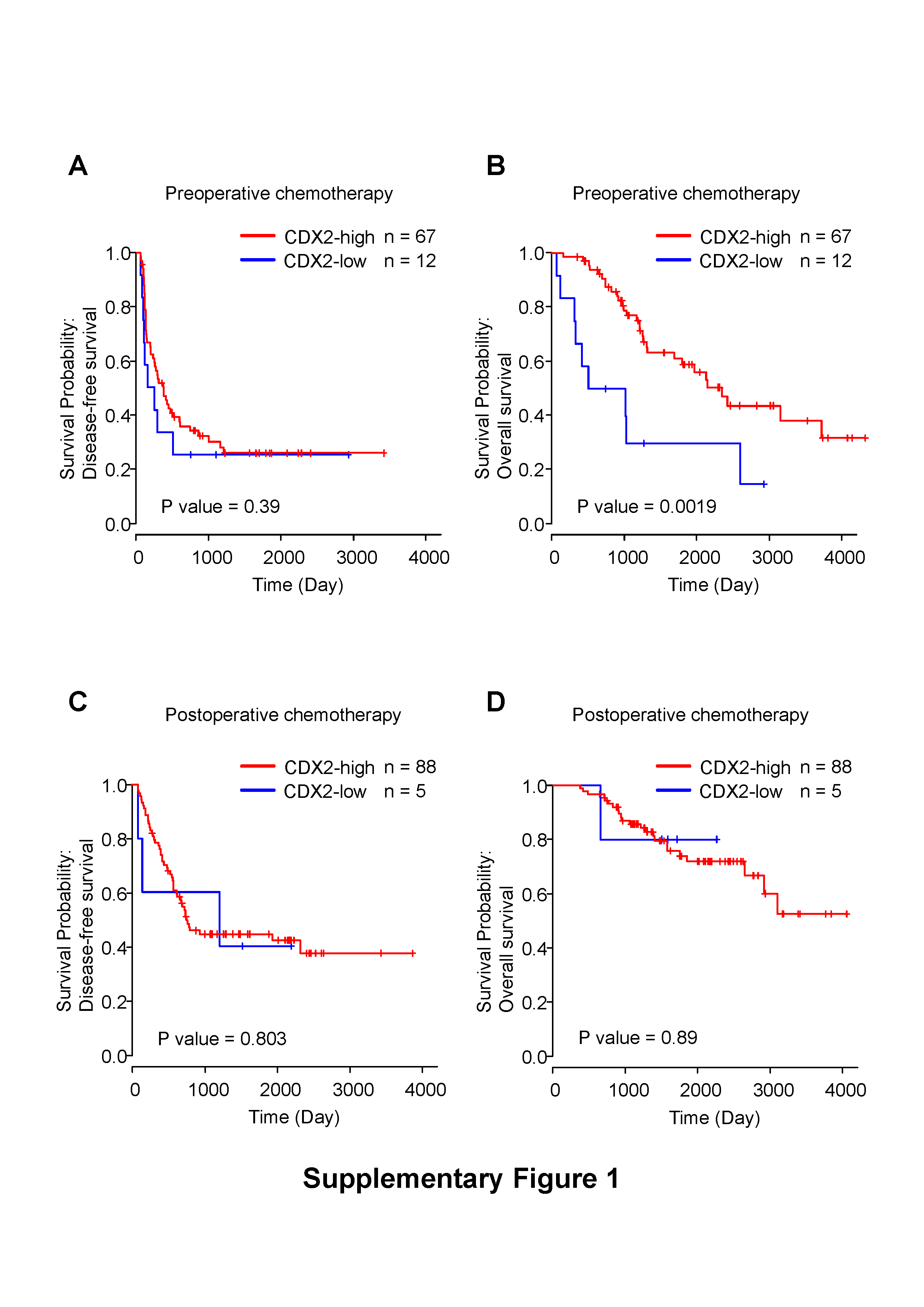

Supplement: Supplementary file 2 — Figure S1. Kaplan-Myere curves of disease-free survival (DFS) and overall survival (OS) in patients undergoing either preoperative or postoperative chemotherapy. (A, B) Those in patients undergoing preoperative chemotherapy. (C, D) Those in patients undergoing postoperative chemotherapy. Blue and red lines represent CDX2-low and CDX2-high CRC, respectively. (TIFF 623 kb) [file 12885_2018_4902_MOESM2_ESM.tiff]
