# Supplementary material for: Impact of CDX2 expression status on the survival of patients after curative resection for colorectal cancer liver metastasis
Source: BMC Cancer. 2018 Oct 16;18:980. doi: 10.1186/s12885-018-4902-8 (PMC6192098; doi:10.1186/s12885-018-4902-8)
Supplement: Supplementary file 3 — Table S2. The relationship between CDX2 expression and overall survival in patients undergoing preoperative chemotherapy. (DOC 80 kb) [file 12885_2018_4902_MOESM3_ESM.doc]

| **Additional file 3: Table S2. The relationship between CDX2 expression and overall survival in patients undergoing preoperative chemotherapy.** | | | | | | |
| --- | --- | --- | --- | --- | --- | --- |
| Features |  | Univariate analysis | |  | Multivariate analysis | |
|  |  | HR (95% CI) | P value |  | HR (95% CI) | P value |
|  |  |  |  |  |  |  |
| CDX2 expression |  |  |  |  |  |  |
| High |  | 1 (reference) |  |  | 1 (reference) |  |
| Low |  | 3.11 (1.46 – 6.62) | 0.003 |  | 3.11 (1.54 – 7.69) | 0.002 |
|  |  |  |  |  |  |  |
| Sex |  |  |  |  |  |  |
| Female |  | 1 (reference) |  |  | 1 (reference) |  |
| Male |  | 1.05 (0.52 - 2.11) | 0.89 |  | 0.86 (0.41 - 1.80) | 0.70 |
|  |  |  |  |  |  |  |
| Tumor grade |  |  |  |  |  |  |
| Well |  | 1 (reference) |  |  | 1 (reference) |  |
| Mod |  | 1.61 (0.57 – 4.57) | 0.36 |  | 1.29 (0.43 – 3.88) | 0.65 |
| Por |  | 12.5 (2.05 - 76.00) | 0.006 |  | 14.20 (2.20 - 91.58) | 0.005 |
|  |  |  |  |  |  |  |
| Tumor location |  |  |  |  |  |  |
| Right |  | 1 (reference) |  |  | 1 (reference) |  |
| Left |  | 0.73 (0.33 - 1.62) | 0.45 |  | 0.80 (0.36 - 1.81) | 0.60 |
| Rectum |  | 0.81 (0.37 - 1.81) | 0.62 |  | 0.86 (0.38 - 1.93) | 0.71 |
|  |  |  |  |  |  |  |
| Liver metastasis |  |  |  |  |  |  |
| Synchronous |  | 1 (reference) |  |  | 1 (reference) |  |
| Metachronous |  | 1.59 (0.84 – 2.98) | 0.15 |  | 1.72 (0.90 - 3.28) | 0.10 |
|  |  |  |  |  |  |  |
