# Supplementary material for: Impact of CDX2 expression status on the survival of patients after curative resection for colorectal cancer liver metastasis
Source: BMC Cancer. 2018 Oct 16;18:980. doi: 10.1186/s12885-018-4902-8 (PMC6192098; doi:10.1186/s12885-018-4902-8)
Supplement: Supplementary file 4 — Table S3. Site of recurrence in patients with CDX2-high and CDX2-low colorectal cancer after potentially curative liver metastasectomy. (DOC 78 kb) [file 12885_2018_4902_MOESM4_ESM.doc]

| **Additional file 4: Table S3. Site of recurrence in patients with CDX2-high and CDX2-low colorectal cancer after potentially curative liver metastasectomy.** | | | | |
| --- | --- | --- | --- | --- |
| Site of recurrence | Total  (n = 260) | CDX2 expression | | P value |
|  |  | High  (n = 232) | Low  (n = 28) |  |
|  |  |  |  |  |
| Liver |  |  |  |  |
| Negative | 67 (25.8) | 63 (27.2) | 4 (14.3) | 0.17 |
| Positive | 193 (74.2) | 169 (72.8) | 13 (85.7) |  |
|  |  |  |  |  |
| Lung |  |  |  |  |
| Negative | 200 (71.4) | 180 (77.6) | 20 (71.4) | 0.48 |
| Positive | 60 (23.1) | 52 (22.4) | 8 (28.6) |  |
|  |  |  |  |  |
| Peritoneum |  |  |  |  |
| Negative | 250 (96.2) | 223 (96.1) | 27 (96.4) | 1.00 |
| Positive | 10 (3.8) | 9 (3.9) | 1 (3.6) |  |
|  |  |  |  |  |
| Adrenal gland |  |  |  |  |
| Negative | 258 (99.2) | 230 (99.1) | 28 (100) | 1.00 |
| Positive | 2 (0.8) | 2 (0.9) | 0 (0) |  |
|  |  |  |  |  |
| Brain |  |  |  |  |
| Negative | 258 (99.2) | 230 (99.1) | 28 (100) | 1.00 |
| Positive | 2 (0.8) | 2 (0.9) | 0 (0) |  |
|  |  |  |  |  |
| Bone |  |  |  |  |
| Negative | 258 (99.2) | 230 (99.1) | 28 (100) | 1.00 |
| Positive | 2 (0.8) | 2 (0.9) | 0 (0) |  |
|  |  |  |  |  |
| Ovary |  |  |  |  |
| Negative | 258 (99.6) | 231 (99.6) | 28 (100) | 1.00 |
| Positive | 1 (0.4) | 1 (0.4) | 0 (0) |  |
|  |  |  |  |  |
| Data presented as n (%) | |  |  |  |
