# Supplementary material for: Impact of CDX2 expression status on the survival of patients after curative resection for colorectal cancer liver metastasis
Source: BMC Cancer. 2018 Oct 16;18:980. doi: 10.1186/s12885-018-4902-8 (PMC6192098; doi:10.1186/s12885-018-4902-8)
Supplement: Supplementary file 5 — Table S4. Association between mismatch repair-deficient and CDX2 expression status in patients with colorectal cancer after potentially curative liver metastasectomy. (DOC 53 kb) [file 12885_2018_4902_MOESM5_ESM.doc]

| **Additional file 5: Table S4. Association between mismatch repair-deficient and CDX2 expression status in patients with colorectal cancer after potentially curative liver metastasectomy.** | | | | |
| --- | --- | --- | --- | --- |
| dMMR status | Total  (n = 396) | CDX2 expression | | P value |
|  |  | High  (n = 360) | Low  (n = 36) |  |
| dMMR negative | 382 (96.5) | 354 (98.3) | 28 (77.8) | < 0.001 |
| dMMR positive | 14 (3.5) | 6 (1.7) | 8 (22.2) |  |
| Data presented as n (%) | |  |  |  |
| Abbreviation: dMMR, mismatch repair deficient | | | | |
