# Supplementary material for: Impact of CDX2 expression status on the survival of patients after curative resection for colorectal cancer liver metastasis
Source: BMC Cancer. 2018 Oct 16;18:980. doi: 10.1186/s12885-018-4902-8 (PMC6192098; doi:10.1186/s12885-018-4902-8)
Supplement: Supplementary file 6 — Table S5. Association between KRAS and CDX2 expression status in patients with colorectal cancer after potentially curative liver metastasectomy. (DOC 53 kb) [file 12885_2018_4902_MOESM6_ESM.doc]

| **Additional file 6: Table S5. Association between *KRAS* and CDX2 expression status in patients with colorectal cancer after potentially curative liver metastasectomy.** | | | | |
| --- | --- | --- | --- | --- |
| *KRAS* status | Total  (n = 174) | CDX2 expression | | P value |
|  |  | High  (n = 160) | Low  (n = 14) |  |
| *KRAS* wild type | 117 (67.2) | 105 (65.6) | 12 (85.7) | 0.14 |
| *KRAS* mutant | 57 (32.8) | 55 (34.4) | 2 (14.3) |  |
| Data presented as n (%) | | | | |
